# Tracking emerging mycotoxins in food: development of an LC-MS/MS method for free and modified *Alternaria* toxins

**DOI:** 10.1007/s00216-018-1105-8

**Published:** 2018-05-16

**Authors:** Hannes Puntscher, Mary-Liis Kütt, Philipp Skrinjar, Hannes Mikula, Joachim Podlech, Johannes Fröhlich, Doris Marko, Benedikt Warth

**Affiliations:** 1 Department of Food Chemistry and Toxicology, Faculty of Chemistry, University of Vienna, Währingerstr. 38, 1090 Vienna, Austria; 2 Institute of Applied Synthetic Chemistry, Vienna University of Technology (TU Wien), Getreidemarkt 9, 1060 Vienna, Austria; 3 Institute of Organic Chemistry, Karlsruhe Institute of Technology, Fritz-Haber-Weg 6, 76131 Karlsruhe, Germany

**Keywords:** *Alternaria alternata*, Masked mycotoxins, Perylene quinones, Liquid chromatography, Tandem mass spectrometry, Food safety

## Abstract

Mycotoxins produced by *Alternaria* fungi are ubiquitous food contaminants, but analytical methods for generating comprehensive exposure data are rare. We describe the development of an LC-MS/MS method covering 17 toxins for investigating the natural occurrence of free and modified *Alternaria* toxins in tomato sauce, sunflower seed oil, and wheat flour. Target analytes included alternariol (AOH), AOH-3-glucoside, AOH-9-glucoside, AOH-3-sulfate, alternariol monomethyl ether (AME), AME-3-glucoside, AME-3-sulfate, altenuene, isoaltenuene, tenuazonic acid (TeA), tentoxin (TEN), altertoxin I and II, alterperylenol, stemphyltoxin III, altenusin, and altenuic acid III. Extensive optimization resulted in a time- and cost-effective sample preparation protocol and a chromatographic baseline separation of included isomers. Overall, adequate limits of detection (0.03–9 ng/g) and quantitation (0.6–18 ng/g), intermediate precision (9–44%), and relative recovery values (75–100%) were achieved. However, stemphyltoxin III, AOH-3-sulfate, AME-3-sulfate, altenusin, and altenuic acid III showed recoveries in wheat flour below 70%, while their performance was stable and reproducible. Our pilot study with samples from the Austrian retail market demonstrated that tomato sauces (*n* = 12) contained AOH, AME, TeA, and TEN in concentrations up to 20, 4, 322, and 0.6 ng/g, while sunflower seed oil (*n* = 7) and wheat flour samples (*n* = 9) were contaminated at comparatively lower levels. Interestingly and of relevance for risk assessment, AOH-9-glucoside, discovered for the first time in naturally contaminated food items, and AME-3-sulfate were found in concentrations similar to their parent toxins. In conclusion, the established multi-analyte method proved to be fit for purpose for generating comprehensive *Alternaria* toxin occurrence data in different food matrices.

**Electronic supplementary material:**

The online version of this article (10.1007/s00216-018-1105-8) contains supplementary material, which is available to authorized users.

**Graphical abstract:**
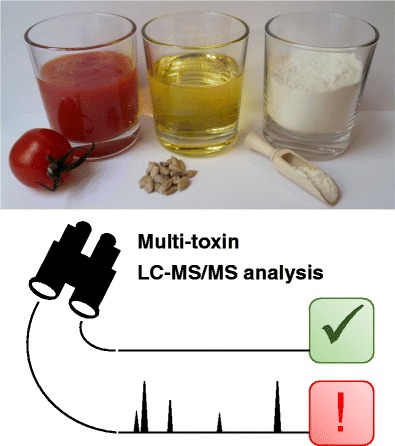
ᅟ

## Introduction

**Fig. 1:**
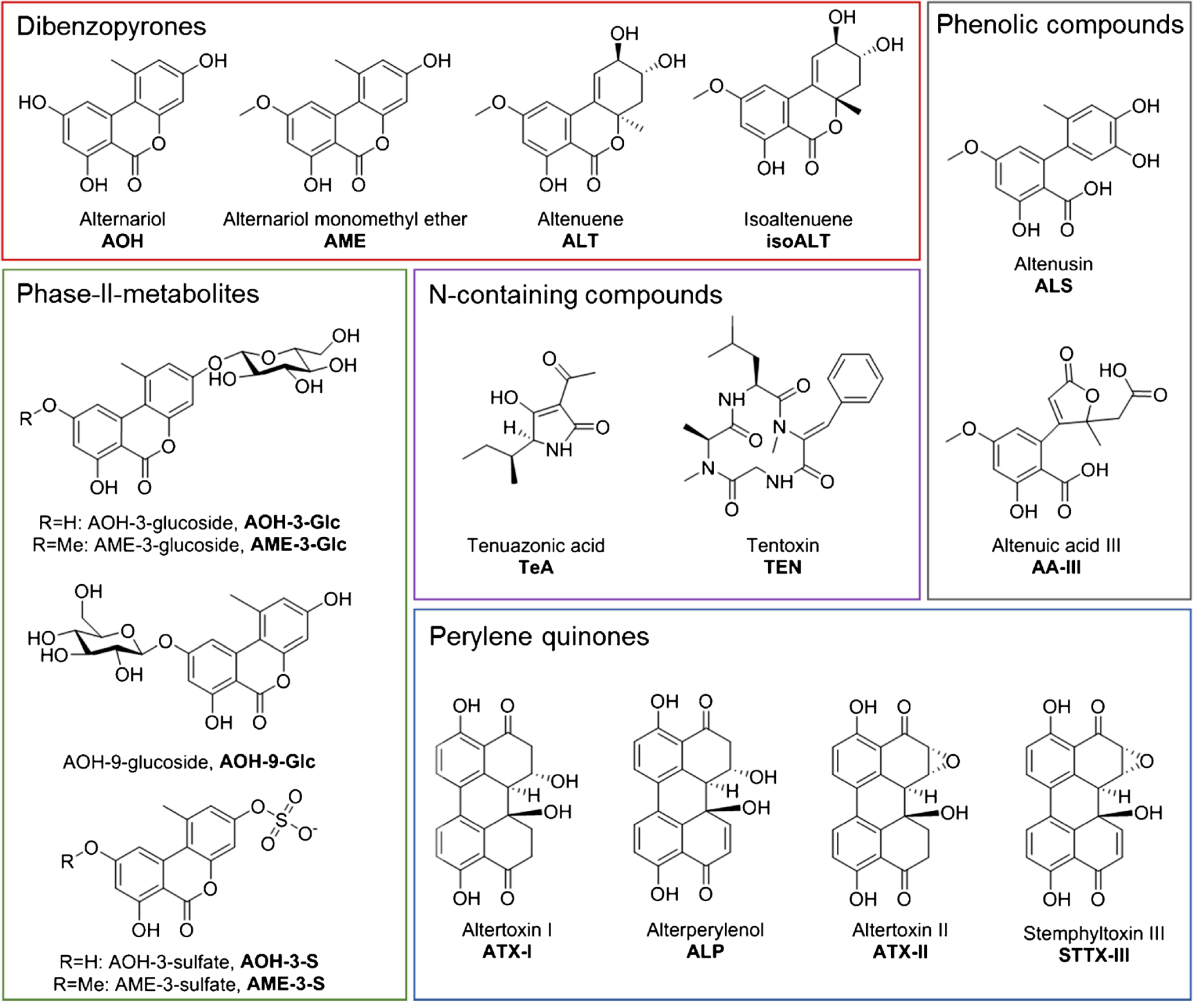
Chemical structures of the 17 *Alternaria* toxins and metabolites included in the developed LC-MS/MS method

*Alternaria* is an ubiquitously occurring fungal genus belonging to the division of *Ascomycota*. About 300 species are known of these so-called black molds, which are considered as both saprophytes and major plant pathogens. *Alternaria* spp. (e.g., *A. alternata*, *A. tenuissima*, *A. solani*, and *A. infectoria*) can infest a wide variety of agricultural crops like cereals (wheat, barley, and sorghum), tomatoes, sunflower seeds, citrus fruits, apples, grapes, and olives [1–5]. Consequences are often considerable economic losses due to crop spoilage or altered visual appearance of the agricultural products. In addition, *Alternaria* strains are capable of producing mycotoxins, toxic secondary metabolites, which can be assigned to five substance classes (Fig. 1): dibenzo-α-pyrone derivatives, e.g., alternariol (AOH), alternariol monomethyl ether (AME), altenuene (ALT), isoaltenuene (isoALT), altenusin (ALS); perylene quinone derivatives, e.g., altertoxin I, II, and III (ATX-I, ATX-II, ATX-III), alterperylenol (ALP), stemphyltoxin III (STTX-III); tetramic acid derivatives, e.g., tenuazonic acid (TeA), allo-tenuazonic acid (alloTeA), altersetin (AST); miscellaneous structures (tentoxin (TEN), altenuic acid III (AA-III); and aminopentol esters, e.g., *A. alternata* f. sp. *Lycopersici* toxins TA1, TA2, TB1, and TB2 (AAL toxins).

Due to the mutagenicity and genotoxicity of some compounds, the contamination of food and feed with *Alternaria* spp. can imply a serious health concern for both humans and animals. TeA is acutely toxic to mice, chicken, and dogs [6]. AOH and AME showed genotoxic and mutagenic effects in vitro [7] and AOH was further demonstrated to poison topoisomerases I and II [8]. ATX-II proved to be an even more potent mutagen causing DNA strand breaks [9, 10]. The mechanisms behind its mode of action could not be clarified so far. Nevertheless, genotoxicity was observed at comparatively low concentrations, but no enhanced levels of reactive oxygen species, glutathione depletion, or topoisomerase inhibition [11, 12]. Besides, AOH, AME, and some of their metabolites additionally exhibit estrogenic potential [13, 14] which may be enhanced by combinatory toxic effects [15–17].

Due to the ability of *Alternaria* spp. to grow even at low temperatures, fungal infestation of agricultural crops and products may also occur post-harvest, even during refrigerated storage or transport [4, 18]. More recently, the European Food Safety Authority (EFSA) released a scientific report on the potential health risks caused by *Alternaria* toxin contaminations of food and elaborated a dietary exposure assessment. The thresholds of toxicological concern (TTC values) were defined as 2.5 ng/kg body weight per day for the genotoxic compounds AOH and AME and 1500 ng/kg body weight per day for non-genotoxic TEN and TeA. Furthermore, EFSA clearly stated the critical need for more comprehensive toxicological characterization and exposure assessment of *Alternaria* toxins to enable a detailed risk assessment [19, 20].

Despite the strict regulatory limits and intensive surveillance features established for a number of major mycotoxins in the European Union and elsewhere, neither legally binding limits nor guidelines are established for *Alternaria* toxins in food or feed to date. An emerging concern in their safety evaluation is the chemical modification of *Alternaria* toxins in the course of plant or animal xenobiotic metabolism [21] or even the metabolism of fungi themselves [22]. Thereby, conjugates of mycotoxins may be formed and referred to as “masked” or “modified” mycotoxins [23].

Liquid chromatography coupled to tandem mass spectrometry has become the technology platform of choice for accurate multi-mycotoxin analysis reaching limits of detection in or even below the nanogram per gram range. Starting from methods targeting only a few *Alternaria* toxins like the AOH and AME [24], additional toxins were included in some methods. Due to the complexity of food matrices, sample preparation strategies like solid phase or QuEChERS extraction were often required to reach satisfying sensitivity [25, 26]. Additionally, the chemical diversity of the target analytes implied chromatographic challenges to overcome. Derivatization of analytes (e.g. TeA) or adapted eluent systems (eluent additives or pH adjustments) helped to improve chromatographic peak shapes [27]. After the total synthesis of masked AOH and AME forms (glucosides and sulfates) [28], these were included into a multi-analyte method to investigate potential contaminations with these selected modified toxins [29, 30]. Walravens et al. [30] investigated 10 *Alternaria* toxins (including the four modified toxins AOH-3-Glc, AOH-3-S, AME-3-Glc, AME-3-S) reaching limits of detection (LOD) between 0.5 and 5 ng/g in cereal-based foodstuffs and in a later publication between 3 and 18.3 ng/g in tomato products [29]. More recent studies included further toxins such as the AAL toxins TA1 and TA2, isoALT [31–33], and altertoxins [5, 34]. Using solid-phase extraction, LODs between 0.1 and 0.6 ng/mL were achieved for 12 *Alternaria* toxins in wine and fruit and vegetable juices [5]. Isotopically labeled internal standards can be used to ensure the performance of quantitative methods and were employed in first applications [30, 35, 36]. The most frequently analyzed food matrices were cereal- and tomato-based products, fruit juices, wine, maize, and sunflower seeds [4, 20, 26, 27, 29, 31–34, 37–40].

The objectives of the study at hand were the development and validation of an LC-ESI-MS/MS method for the simultaneous detection and quantitation of the most relevant *Alternaria* mycotoxins in food. Twelve parent compounds as well as five modified toxins, partly assessed for the first time, were included to allow for the broadest coverage of *Alternaria* toxins reported in literature. The method validation was performed for three highly diverse matrices, namely tomato sauce, sunflower seed oil, and wheat flour. To demonstrate the applicability of the method and to gain first insights on *Alternaria* toxin contaminations in food available on the Austrian retail market, a small-scale survey was performed.

## Materials and methods

### Reagents, solvents, and chemicals

Commercially available reference materials were purchased from the following sources: TeA and TEN from Sigma-Aldrich (Steinheim, Germany), AOH and AME from Toronto Research Chemicals (Ontario, Canada), and ALS from Eubio (Vienna, Austria). ALT, isoALT, and AA-III were synthesized at the Institute of Organic Chemistry (Karlsruhe Institute of Technology, Germany) [41, 42], while AOH-3-Glc, AOH-9-Glc, AOH-3-S, and AME-3-Glc were synthesized at the Institute of Applied Synthetic Chemistry (Vienna University of Technology (TU Wien), Vienna, Austria) [28]. ATX-I, ATX-II, STTX-III, and ALP were isolated from fungal cultures grown on rice by an optimized protocol based on Schwarz et al. [10]. Methanol (MeOH), water, and acetonitrile (ACN, LC-MS grade) were purchased from Honeywell (Seelze, Germany); ammonia solution (25% in water, for LC-MS) and ammonium acetate (NH_4_Ac, LC-MS grade) were purchased from Sigma-Aldrich. For sample preparation, Milli-Q water, MeOH (HPLC grade), and acetic acid (p.a.) from Sigma-Aldrich (Steinheim, Germany) and *n*-hexane (p.a.) from Carl Roth GmbH (Karlsruhe, Germany) were used.

Stock solutions of reference standards were prepared by dissolving solid substances to a final concentration of 10–500 μg/mL in MeOH. Mycotoxin conjugates (AOH-3-Glc, AOH-9-Glc, AOH-3-S, AME-3-Glc, AME-3-S) were dissolved in water/ACN (20/80, *v*/*v*). Working solutions containing all analytes at a concentration of 2.5–12.5 μg/mL were prepared in MeOH by diluting the individual stock solutions. Working solutions were prepared freshly after 4 weeks. Reference standards and the prepared solutions were stored at − 20 °C.

### Sample preparation

Homogenized samples (1.000 ± 0.005 g) were extracted with 5 mL of extraction solvent (MeOH/water/HAc, 79/20/1, *v*/*v*/*v*) for 60 min using an overhead shaker (Roto-Shake Genie, Scientific Industries, NY, USA). The addition of *n*-hexane (1 mL) to sunflower seed oil samples before shaking lowered the viscosity and improved homogenization and extraction. Subsequently, extracts were diluted 1:1 with MeOH/water (10/90, *v*/*v*) after removing the *n*-hexane layer in case of sunflower seed oil samples. The diluted extracts were centrifuged at 20,000 rcf and 4 °C for 15 min. Flour samples were additionally filtered using a syringe filter (Cameo™, PTFE, 0.22 μm pore size, Carl Roth, Germany), since centrifugation did not remove fine particles sufficiently.

### LC-MS/MS parameters and analysis

Sample analysis was performed on a high-performance liquid chromatography system (UltiMate3000) connected to a triple-quadrupole mass spectrometer (TSQ Vantage) equipped with a heated electrospray ionization interface (all from Thermo Scientific). Chromatographic separation was realized on a Supelco Ascentis Express column (C18, 2.7 μm, 10 cm × 2.1 mm) by a binary gradient elution at a flow rate of 0.4 mL/min. The column was equipped with a Phenomenex SecurityGuard™ precolumn (C18, 2 mm). Eluent A was an NH_4_Ac solution in water (5 mM, pH adjusted to 8.7 with 25% ammonia solution), while MeOH was used as eluent B. The multi-step gradient was optimized in order to baseline separate even analyte isomers as follows: During the first minute, the column was kept at 10% eluent B, before raising to 38% within half a minute. Subsequently, the percentage of eluent B was linearly raised to 40% until minute 6, to 58% until minute 6.1, to 61% until minute 7.5, and to 85% until minute 9. Then, an isocratic column-purging phase at 100% eluent B (from 9.1 to 13 min) was followed by 2 min of equilibration at initial conditions. Overall, this resulted in a run time of 15.5 min. A volume of 5 μL was injected onto the column. The autosampler compartment and the column oven temperature were maintained at 10 and 30 °C, respectively. A divert valve was utilized to direct the effluent to the waste between 0.5 and 1.5 min.

The mass spectrometer was operated in multiple reaction monitoring (MRM) mode using negative ionization, which was switched to positive mode for the last 2.5 min of each run to prevent potential charging effects. Ion source (ESI) parameters were optimized as follows: spray voltage − 3000 V; vaporizer temperature 400 °C; sheath gas pressure 35 Arb; ion sweep gas pressure 5 Arb; auxiliary gas pressure 20 Arb; capillary temperature 325 °C. The MS and MS/MS parameters were optimized by direct injection of reference standards and are reported in Table 1.

**Table 1:** Mass spectrometric parameters and analyte specific retention times as optimized by direct infusion experiments and obtained during method validation

Analytes	RTs	Parent ion		S-Lens	Product ions				Ion ratio^a^
					Quantifier		Qualifier		
	[min]	[*m/z*]		[V]	[*m/z*]	CE [V]	[*m/z*]	CE [V]	[%]
AOH	6.6 ± 0.3	257	[M-H]-	70	215	27	147	33	44 ± 2
AME	10.0 ± 0.0	271	[M-H]-	73	256	23	227	38	17 ± 0.4
ALT	6.9 ± 0.0	291	[M-H]-	76	229	18	248	20	52 ± 13
isoALT	7.3 ± 0.0	291	[M-H]-	76	203	32	248	20	78 ± 4
TeA	2.1 ± 0.1	196	[M-H]-	88	139	22	112	26	51 ± 8
TEN	8.9 ± 0.0	413	[M-H]-	100	271	19	141	22	88 ± 3
AOH-3-Glc	3.5 ± 0.1	419	[M-H]-	101	256	31	228	42	23 ± 1
AOH-9-Glc	4.8 ± 0.0	419	[M-H]-	101	256	31	228	42	25 ± 3
AOH-3-S	3.7 ± 0.1	337	[M-H]-	86	257	22	213	37	14 ± 1
AME-3-Glc	8.5 ± 0.0	433	[M-H]-	104	270	34	227	44	76 ± 5
AME-3-S	8.0 ± 0.0	351	[M-H]-	88	256	34	271	22	60 ± 1
ATX-I	8.5 ± 0.0	351	[M-H]-	73	315	18	333	14	65 ± 4
ATX-II	9.5 ± 0.0	349	[M-H]-	88	285	34	332	15	26 ± 1
ALP	8.6 ± 0.0	349	[M-H]-	68	303	18	261	28	90 ± 3
STTX-III	9.7 ± 0.0	347	[M-H]-	71	329	19	301	29	168 ± 26
ALS	3.7 ± 0.0	289	[M-H]-	76	245	18	230	22	39 ± 2
AA-III	2.6 ± 0.3	321	[M-H]-	82	233	17	189	22	74 ± 1

^a^The ion ratio (quantifier/qualifier*100) in spiked samples was calculated as an average of the values obtained for the three matrices

Multi-analyte solutions, both in neat solvent and matrix, were obtained by diluting the working solutions to eight concentration levels covering three orders of magnitude. Linear regressions were weighted by a factor of 1/x. Due to matrix effects, matrix-matched calibration was used for quantitation (see Electronic Supplementary Material (ESM), Figs. S1, S2, and S3). During longer sequences, the calibration set was repeated after every 20 sample injections to account for potential intensity shifts. Solvent standards were measured before and after every sequence as an additional QC measure. Moreover, solvent and matrix blanks were regularly injected. The general integrity of the instrumentation was confirmed before and after every sequence by the measurement of a QC reference standard mix in triplicate by evaluating retention times, peak shapes, and areas. Chromeleon™ Chromatography Data System Software (version 6.80 SR13 Build 3818) and Xcalibur™ Software (version 3.0, Thermo Scientific) were used for instrument control and data acquisition. Data evaluation was performed with TraceFinder™ (version 3.3) and parameters for compound optimization were tuned using Thermo TSQ Tune Master (version 2.5.0.1305).

### Validation experiments

Since certified reference materials for the analysis of *Alternaria* toxins are not commercially available to date, the development and validation of the presented method were based on the fortification of blank matrix samples with reference standards. Chromatographic peaks of the quantifier and qualifier ions of the 17 analytes in spiked tomato sauce are shown in the ESM (Fig. S4). According to Commission Decision (EC) No. 657/2002 [43] and the Eurachem Laboratory Guide for the validation of analytical methods [44], the method validation for the three matrices was evaluated by the following parameters: selectivity, linearity, matrix effects, recovery, limit of detection (LOD) and quantitation (LOQ), repeatability (intraday precision, RSD_r_), and intermediate precision (interday precision, RSD_R_).

According to the Commission Decision (EC) No. 657/2002, “concerning the performance of analytical methods and the interpretation of results”, four identification points were considered for each analyte. The chromatographic peak area of the product ion with the most favorable intensity and signal-to-noise ratio properties was used for quantitation (quantifier MRM parent-to-product transition). A second product ion chromatogram (qualifier MRM parent-to-product transition) was used to confirm the identity of the signal as well as the ratio between these two transitions (relative ion intensity). Furthermore, the retention time (RT) was compared to the reference standard [43].

The selectivity was investigated by the analysis of blank matrix samples for each matrix. The chromatograms were compared to artificially fortified (spiked) blank matrix samples to ensure the absence of interfering peaks. The linearity of the calibration curves (5–7 concentration levels in solvent and matrix-matched) was evaluated by the regression coefficient (*R*^2^). Signal suppression and enhancement (SSE) caused by matrix effects was calculated as the ratio of calibration curve slopes of each analyte in the respective matrix and in neat solvent. The relative recoveries (*R*_*E*_ in %; equal to extraction efficiency) were calculated as the ratio of analyte concentration quantified using the matrix-matched calibration curve and the known spiking level in the fortified blank samples. For this purpose, analysis of samples spiked at three levels (low, medium, high) was performed in triplicate for all food matrices. The in-house validation was carried out over a duration of three consecutive weeks in triplicate (independent sample preparation, extraction, and analysis) by two operators.

To determine the LOD and LOQ values, measurements of blank samples spiked at the lowest concentration level and the matrix-matched calibration solutions were examined. For each individual analyte in a specific matrix, LOD and LOQ values were determined by manual estimation of the analyte’s signal-to-noise ratio of three and six, respectively. The repeatability (intraday precision, RSD_r_) and intermediate precision (interday precision, RSD_R_) were calculated from the standard deviations of the samples spiked at three levels as described above and measured twice within the same sequence on three different days during 3 consecutive weeks.

### Collection of retail samples

Food commodities (tomato sauces (*n* = 12), sunflower seed oils (*n* = 7), and wheat flowers type 480 (*n* = 9)) were collected from different supermarket stores in Vienna, Austria, between March and April 2017. A homogenized, aggregate sample of at least 1 kg was prepared according to the Commission Regulation (EC) No. 2006/401, “laying down the methods of sampling and analysis for the official control of the levels of mycotoxins in foodstuffs” [45]. Laboratory samples of 50–100 g were stored (tomato sauces at − 20 °C, sunflower seed oils at 4 °C, and wheat flower samples at room temperature) until sample preparation and LC-MS/MS analysis.

## Results and discussion

### Optimization of MS and MS/MS parameters

The optimization of the electrospray ionization interface and the tandem-mass-spectrometric parameters were achieved by infusions of reference material solutions (individual analytes in MeOH, 5 μg/mL) using a syringe pump. To mimic actual infusion conditions, a flow rate of 3–10 μL/min tune solution was combined with an LC flow of 0.3 mL/min using a T-piece before entering the ESI source. During the infusion optimization, the ratio of eluent A and B was selected according to the polarity of a certain analyte (i.e., highly polar analytes were optimized at between 10 and 40% eluent B and lipophilic analytes between 60 and 90% eluent B). MS and MS/MS parameters were optimized in both polarities using the instruments’ tuning software. First, the most appropriate precursor ions were selected by maximum intensity in full scan mode. Interestingly, negative ionization led to higher signals for all 17 target analytes. The eight most abundant product ions and their corresponding collision energies were determined for each analyte by comparing signal intensities with changing parameters. To achieve highest sensitivity and selectivity in the three food matrices, S/N ratios for all eight transitions were evaluated by independent LC-MS injections of spiked blank matrix samples. The two ions with the highest relative S/N value, which were fortunately identical in all matrices, were selected as quantifier and qualifier ion (Table 1). ESI parameters (spray voltage, vaporizer temperature, sheath gas, ion sweep gas, auxiliary gas, and capillary temperature) were examined manually and set to provide the best overall performance. It was suggested that charging effects in an ESI interface may result in signal suppression of certain challenging analytes including TeA [5]. Hence, the ionization polarity was switched from negative to positive mode for the final 2.5 min of each run.

### Development of the chromatographic method

**Fig. 2:**
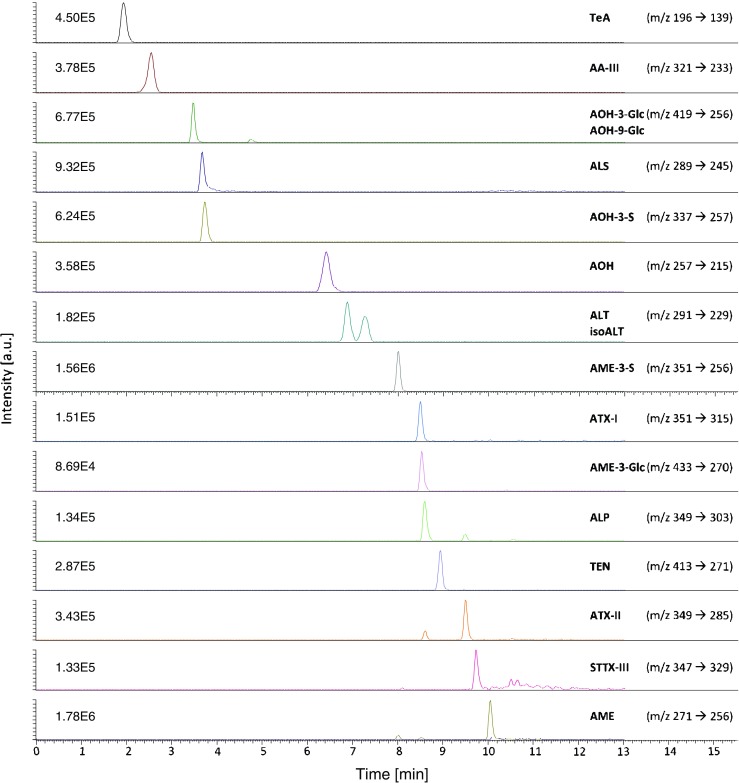
Chromatographic separation of the multi-component standard (highest level of the linear range, see Table 2) containing the 17 target analytes

An important aim of the developed chromatographic method was to baseline-separate the isomeric analytes of interest in reasonable run times resulting in reproducible, sharp, and symmetrical signals. The selected reversed phase column was described before to be favorable for the separation of the five *Alternaria* toxins, namely AOH, AME, ALT, TeA, and TEN [27]. By optimization of eluents, temperature, and the multi-step gradient, we were able to separate our 17 target analytes of highly diverse polarity. A basic eluent system (eluent A, 5 mM NH_4_Ac in water, pH 8.7) was crucial for a symmetric peak shape of the polar TeA, which is typically measured after derivatization or alternatively exhibits very broad peaks and peak tailing in acidic eluents. Also, the AOH-3-S and AME-3-S showed peak tailing with acidic eluents, which could be resolved using the basic conditions. Importantly, also, the most lipophilic toxins (AME, TEN, and the perylen quinones) showed a favorable behavior with very narrow peak shapes allowing for enhanced signal intensities. Due to the optimized multi-step gradient, it was possible to baseline-separate the isomers ALT and isoALT, as well as the glucosides of AOH for the first time (Fig. 2). Purging of the column after finishing measurement sequences at 95% MeOH ensured an acceptable column lifetime despite the applied basic eluents. The flow rate and column temperature were optimized to yield the overall best signal-to-noise ratios and shortest run time. In general, retention times of the target analytes were stable and reproducible (see Table 1). Only the retention times of TeA, AA-III, and AOH and its modified forms were prone to minor pH changes observed after preparing fresh eluent A. Maximum shifts have been observed for TeA (0.1 min), AOH (0.3 min), and AA-III (0.3 min). However, since signals derived from reference standards and unknown samples behave equally, this was not an issue. No relevant carry-over between injections was observed. However, the absence was constantly verified by monitoring solvent and matrix blank samples. The injection needle of the LC autosampler was washed with 100 μL isopropanol/water (75/25, *v*/*v*) before and after each sample injection.

### Optimization of the sample preparation protocol

The sample preparation protocol was intended on one hand to be as generic as possible to prevent the discrimination of any of the chemically diverse analytes and, on the other hand, to be time- and cost-effective. Hence, sample extracts were centrifuged and diluted by a factor of two resulting in an overall dilution of 1:10 (*w*/*v*). Since the LC-MS/MS method was thoroughly optimized and allows for highly sensitive and selective quantitation, no further derivatization [27] or solid-phase extraction [5, 27] steps were required. This makes the method attractive for large-scale food-monitoring programs as suggested by the EFSA in their recent scientific report [20]. Due to fine particles suspended in the wheat flour extracts, an additional filtration prior to analysis was required. This ensured reproducible pressure conditions of the LC system even after a high number of injections. The addition of *n*-hexane to the sunflower seed oil samples simplified their handling and led to enhanced extraction efficiency. This was not necessary for tomato sauce and wheat flower samples.

### Method validation

In-house validation was performed based on the requirements defined by the Commission Decision (EC) No. 657/2002 [43] and the Eurachem Laboratory Guide for the validation of analytical methods [44]. Three food commodities with diverse chemical composition and frequently contaminated by *Alternaria* mycotoxins [1, 3, 46–48] were chosen for comprehensive evaluation of the developed method and included tomato sauce (representative for aqueous matrices), sunflower seed oil (non-polar and fatty matrices), and wheat flour (carbohydrate-based matrices). The following parameters were successfully validated: selectivity linearity, matrix effects, recovery, sensitivity, repeatability, and intermediate precision. Due to a lack of certified reference materials of *Alternaria* toxins, the validation was based on the fortification of blank matrix samples at three concentration levels. These concentrations were based on the preliminary calculation of LOQ values (Table 2).

**Table 2:** Method validation parameters including spiking levels, limits of detection (LOD) and quantitation (LOQ), linear range, extraction efficiency (R_E_), intermediate precision (RSD_R_) and repeatability (RSD_r_), as well as signal suppression and enhancement (SSE)

Analytes	Spiking levels^a^	LOD^b, c^	LOQ^d, c^	Range^e^	R_E_^f^ ± RSD_R_^g^			RSD_r_^h^			SSE^i^		
	[ng/g]	[ng/g]	[ng/g]	[ng/mL]	[%]			[%]			[%]		
					T	O	F	T	O	F	T	O	F
AOH	5/25/125	0.5/0.1/0.6	1.0/0.2/1.2	0.05–100	87 ± 11	95 ± 15	94 ± 11	15/4/3	35/11/3	29/10/4	94	102	95
AME	1/5/25	0.05/0.03/0.05	0.1/0.06/0.1	0.01–20	86 ± 10	84 ± 25	99 ± 11	9/3/5	20/6/5	11/6/3	81	93	124
ALT	30/150/750	5.0/5.5/6.0	10/11/12	0.3–600	77 ± 15	91 ± 13	94 ± 13	22/15/4	22/15/8	14/8/8	99	109	112
isoALT	30/150/750	3.5/3.0/4.0	7/8/9	0.3–600	78 ± 24	83 ± 17	96 ± 16	23/32/4	30/8/3	21/8/6	101	105	99
TeA	60/300/1500	6/4/7	12/8/14	0.6–1200	77 ± 13	75 ± 12	91 ± 15	22/11/6	16/8/5	19/10/6	90	98	83
TEN	1.5/7.5/37.5	0.1/0.05/0.5	0.2/0.1/1.0	0.015–30	84 ± 19	98 ± 25	92 ± 21	34/18/8	33/8/6	19/21/7	90	111	83
AOH-3-Glc	5/25/125	1.0/0.8/1.0	2.0/1.6/2.0	0.05–100	81 ± 20	94 ± 16	94 ± 16	28/14/5	32/9/3	23/8/3	51	118	68
AOH-9-Glc	20/100/500	4.5/8.0/6.0	9/16/12	1.2–400	85 ± 44	83 ± 29	86 ± 27	19/38/17	7/28/16	42/39/12	79	114	72
AOH-3-S	5/25/125	1.0/1.8/1.0	2.0/3.6/2.0	0.05–100	82 ± 12	91 ± 15	55 ± 20	19/9/3	28/10/5	31/15/7	84	101	97
AME-3-Glc	10/50/250	2.0/2.0/1.0	4.0/4.0/2.0	0.2–200	82 ± 34	93 ± 25	87 ± 38	19/23/12	40/12/11	53/30/5	97	104	103
AME-3-S	5/25/125	0.05/0.05/0.1	0.1/0.1/0.2	0.05–100	84 ± 12	100 ± 9	59 ± 12	9/5/4	8/6/3	16/4/3	107	95	156
ATX-I	1/5/25	0.2/0.5/0.6	0.4/1.0/1.2	0.02–20	79 ± 22	92 ± 20	84 ± 29	16/21/16	48/20/8	83/15/11	103	102	89
ATX-II	5/25/125	0.5/1.5/1.2	1.0/3.0/2.4	0.05–100	81 ± 14	80 ± 19	83 ± 23	34/10/10	32/8/8	29/28/8	101	102	96
ALP	5/25/125	0.8/1.0/1.0	1.6/2.0/2.0	0.05–100	75 ± 21	89 ± 24	79 ± 28	33/13/12	28/18/6	45/22/4	103	103	97
STTX-III	5/25/0	0.5/3.0/1.0	1.0/6.0/2.0	0.3–10	51 ± 49	94 ± 32	28 ± 71	31/29/–	22/19/–	75/19/–	106	113	144
ALS	30/150/750	1.8/8.0/9.0	3.6/16.0/18.0	0.3–600	77 ± 9	97 ± 20	19 ± 29	11/5/2	23/18/7	–/13/22	144	14	56
AA-III	10/50/250	1.5/1.5/1.8	3.0/3.0/3.6	0.1–200	83 ± 14	88 ± 11	64 ± 26	10/9/7	15/10/4	23/16/12	104	106	131

^a^Fortification levels were identical for the three matrices (tomato sauce, T; sunflower seed oil, O; wheat flour, F) chosen for low/middle/high concentrations

^b^Limit of detection

^c^Values in the following order: tomato sauce/sunflower seed oil/wheat flour matrix

^d^Limit of quantitation

^e^Concentration range with linear regression

^f^Relative recovery (extraction efficiency), calculated as the ratio of analyte concentration quantified using the matrix-matched calibration curve and the known spiking level in the fortified blank samples, averaged over the results of the three spiking levels

^g^Intermediate precision (interday precision), calculated from the standard deviations of the samples spiked at three levels and measured on three different days of the three consecutive weeks, averaged over the results of the three spiking levels

^h^Repeatability (intraday precision), calculated from the standard deviations of the samples spiked at three levels measured twice within the same sequence

^i^Signal suppression and enhancement, calculated as the ratio of calibration curve slopes of the respective matrix and neat solvent

Selectivity was verified by the analysis of representative blank samples for each matrix. Signals from fortified blank samples and unknown samples collected from the Austrian retail marked were evaluated and no relevant co-eluting interfering signals were detected. For all analytes, suitable blank matrix samples were identified with the exception of TEN in sunflower seed oil in which all samples contained very low concentrations (see Table 3). TEN was clearly identified in these oils, seeing that this analyte proved very stable retention times (in all matrixes), reproducible narrow peak shapes, and a low background noise. The sample contaminated with the lowest amount of TEN (< LOQ) was used in the spiking experiments. The regression coefficients (*R*^2^) between 0.97 and 0.99 confirmed linearity of both solvent- and matrix-matched calibration curves over at least 3 orders of magnitude (5–7 concentration levels, Table 2).

**Table 3:** Results of a pilot survey to determine *Alternaria* toxins in food samples purchased in Austria: tomato sauces (*n* = 12), sunflower seed oils (*n* = 7), wheat flours (*n* = 9). Eight of the 17 analytes included in the method were not detected in any sample and are thus not reported in the table. Abbreviations: *n.d*. not detected

		Origin	Cultivation	AOH	AME	isoALT	TeA	TEN	AOH-3-Glc	AOH-9-Glc	AOH-3-S	AME-3-S
Tomato sauce	1	Italy	Conventional	n.d.	n.d.	n.d.	42	n.d.	n.d.	< LOQ	n.d.	n.d.
	2	Italy	Conventional	< LOQ	n.d.	n.d.	117	n.d.	n.d.	n.d.	n.d.	< LOQ
	3	Italy	Conventional	n.d.	n.d.	n.d.	< LOQ	n.d.	n.d.	n.d.	n.d.	n.d.
	4	Morocco	Conventional	n.d.	n.d.	n.d.	n.d.	n.d.	n.d.	n.d.	n.d.	n.d.
	5	Italy	Organic	20.2	4.0	n.d.	323	< LOQ	n.d.	12.7	< LOQ	3.2
	6	Italy	Organic	n.d.	< LOQ	< LOQ	125	< LOQ	n.d.	n.d.	n.d.	<LOQ
	7	Italy	Organic	< LOQ	n.d.	n.d.	114	< LOQ	n.d.	n.d.	n.d.	n.d.
	8	Spain	Organic	n.d.	n.d.	n.d.	n.d.	n.d.	n.d.	n.d.	n.d.	n.d.
	9	Italy	Organic	1.4	< LOQ	n.d.	72	< LOQ	< LOQ	n.d.	< LOQ	1.4
	10	Italy	Organic	< LOQ	< LOQ	n.d.	233	0.6	n.d.	n.d.	n.d.	< LOQ
	11	Italy	Organic	< LOQ	n.d.	n.d.	< LOQ	n.d.	n.d.	n.d.	n.d.	n.d.
	12	Italy	Organic	< LOQ	< LOQ	n.d.	65	n.d.	n.d.	n.d.	n.d.	< LOQ
Sunflower seed oil	1	Austria	Conventional	n.d.	n.d.	n.d.	n.d.	< LOQ	n.d.	n.d.	n.d.	n.d.
	2	Austria	Conventional	< LOQ	2.2	n.d.	n.d.	< LOQ	n.d.	n.d.	n.d.	n.d.
	3	Austria	Conventional	n.d.	< LOQ	n.d.	n.d.	< LOQ	n.d.	n.d.	n.d.	n.d.
	4	Germany	Organic	< LOQ	1.7	n.d.	29	3.4	n.d.	n.d.	n.d.	n.d.
	5	Austria	Organic	0.5	< LOQ	n.d.	25	1.8	n.d.	n.d.	n.d.	n.d.
	6	Germany	Organic	n.d.	n.d.	n.d.	< LOQ	< LOQ	n.d.	n.d.	n.d.	n.d.
	7	Germany	Organic	n.d.	0.7	n.d.	21	< LOQ	n.d.	n.d.	n.d.	n.d.
Wheat flour	1	Austria	Conventional	n.d.	n.d.	n.d.	n.d.	n.d.	n.d.	n.d.	n.d.	n.d.
	2	Austria	Conventional	n.d.	n.d.	n.d.	n.d.	n.d.	n.d.	n.d.	n.d.	n.d.
	3	Austria	Conventional	n.d.	n.d.	n.d.	n.d.	< LOQ	n.d.	n.d.	n.d.	n.d.
	4	Austria	Conventional	n.d.	n.d.	n.d.	n.d.	n.d.	n.d.	n.d.	n.d.	n.d.
	5	Austria	Conventional	n.d.	n.d.	n.d.	n.d.	n.d.	n.d.	n.d.	n.d.	n.d.
	6	Austria	Organic	n.d.	n.d.	n.d.	n.d.	n.d.	n.d.	n.d.	n.d.	n.d.
	7	Austria	Organic	n.d.	n.d.	n.d.	n.d.	n.d.	n.d.	n.d.	n.d.	n.d.
	8	Austria	Organic	< LOQ	n.d.	n.d.	n.d.	n.d.	n.d.	n.d.	n.d.	n.d.
	9	Austria	Organic	n.d.	n.d.	n.d.	n.d.	n.d.	n.d.	n.d.	n.d.	n.d.

Matrix effects varied depending on the type of matrix as reported in Table 2. Signal suppression or enhancement (SSE) for AOH, ALT, isoALT, TeA, TEN, AOH-3-S, AME-3-Glc, ATX-I, ATX-II, and ALP was between 80 and 120%. AME and its sulfate showed a signal enhancement in wheat flour of 124 and 156%, respectively. Sulfate conjugates of other mycotoxins have been described to be prone to signal enhancement before [49]. Signals of AOH-3-Glc and AOH-9-Glc were suppressed in tomato sauces (51 and 79%) and wheat flour (68 and 72%), but enhanced in sunflower seed oil (118 and 114%). Signal enhancement in wheat flour was also found for STTX-III and AA-III. ALS showed to be susceptible to matrix effects with 144% in tomato sauce and 14 and 56% in sunflower seed oil and wheat flour, respectively, for its parent ion [M-H]^−^ at *m/z* 289. Previous methods did not determine the deprotonated parent ion, but an ion at *m/z* 287, which may represent a ring-closing reaction product [5, 50]. This ion of unknown structure shows lower matrix effects; however, it also yielded lower signal-to-noise ratios and thus significantly higher LOD values. Therefore, we selected the [M-H]^−^ ion for the final method.

The relative recovery (*R*_*E*_, extraction efficiency) of most analytes ranged between 70 and 110% in all three matrices, a range comparable with other methods published in literature [5, 27, 30]. Best results were obtained for sunflower seed oil, where the values ranged between 74 and 100% for all three spiking levels. The extraction proved to be very suitable for tomato sauce as well, only the recovery of STTX-III was below the target value. Wheat flour was a comparatively more challenging matrix. The more polar analytes AA-III, AOH-3-S, and AME-3-S exhibited recoveries between 55 and 64%, while it was even lower for ALS. Recoveries for AOH-3-S and AME-3-S in cereal-based food items published by Walravens et al. [30] were close to 100%, but no recoveries for AA-III and ALS in similar matrices were reported so far. Apparently, molecules holding deprotonable sulfate or carboxyl groups are less effectively extracted from wheat flour with the utilized extraction procedure. Recoveries of STTX-III, which are the first reported for any food matrix, were 94% in sunflower seed oil, but only 28–51% in tomato sauce and wheat flour. Due to the limited amounts available of the reference standard, no further investigations could be performed. Consequently, accurate quantitation of this analyte in two matrices (tomato sauces and wheat flour) is not possible but the analyte was kept in the final method for semi-quantitative assessment. Since this analyte was never determined in any food commodity before, to the best of our knowledge, it may enable first indications of the presence of this potentially potent toxin holding an epoxide group [51, 52].

The limits of detection (LODs) of the presented method were between 0.03 ng/g (AME) and 7 ng/g (ALS), whereas the limits of quantitation (LOQ) were between 0.06 ng/g (AME) and 19 ng/g (ALS). Key toxins including AOH and ATX-II can be detected down to 1 ng/g, in the case of AME and TEN even down to 0.1 and 0.5 ng/g, respectively. For TeA, which was indicated as a challenging analyte before [5, 27], but frequently occurs at higher concentrations in food stuff, an LOD of 6 ng/g in tomato sauce, 4 ng/g in sunflower seed oil, and 7 ng/g in wheat flour was achieved. Moreover, the modified mycotoxins (AOH-3-Glc, AOH-9-Glc, AOH-3-S, AME-3-Glc, and AME-3-S) can be detected as low as 0.05–6 ng/g. The repeatability (intraday precision, RSD_r_) and intermediate precision (interday precision, RSD_R_) proved to be satisfying for nearly all analytes and matrices. Even though the presented method’s sample preparation does not include derivatization or solid-phase extraction steps, compared to earlier published studies, LOD values reached a similar or even lower range for most analytes [5, 27, 30]. For AME, TEN, ATX-I, AOH-3-S, AME-3-Glc, and AME-3-S, lower LODs were achieved compared to Zwickel et al. [5], Walravens et al. [30], while for TeA and ALT, they were slightly higher. Due to the shortage of reference materials for perylene quinones (ATX-I, ATX-II, STTX-III, ALP), modified forms of AOH and AME or toxins like iso-ALT, ALS, and AA-III, there is not much data available in literature about these compounds. In conclusion, the performed validation demonstrated that the newly developed method is fit for purpose, generating valuable occurrence data of up to 17 *Alternaria* toxins for the first time simultaneously.

### Application to naturally contaminated food samples

To gain first insights on contamination levels and patterns of *Alternaria* toxins including modified forms, samples from the Austrian market (*n* = 28) were analyzed in a small-scale survey. Three independent measurements of tomato sauce (*n* = 12), sunflower seed oil (*n* = 7), and wheat flower (*n* = 9) samples were performed and average values are reported in Table 3.

Overall, nine of the 17 toxins included in the developed method have been determined in products intended for human consumption. This is intriguing given the rather small sample number analyzed in this preliminary study. In future large-scale occurrence surveys, or when analyzing visually mold-infested samples, it is likely to observe even a greater number of these emerging contaminants.

Tomato sauce was the commodity with both the highest number of detected analytes and generally the highest concentrations. This is in line with literature suggesting tomato-based products to be often contaminated by comparatively high levels of the four to six typically reported *Alternaria* toxins AOH, AME, TeA, TEN, ALT, and ATX-I [2, 4, 20, 27, 29, 31, 37]. Interestingly, organic products seem to be slightly more contaminated than conventionally farmed samples. However, this should not be over-interpreted due to the limited sample size but investigated in more detail in further studies.

**Fig. 3:**
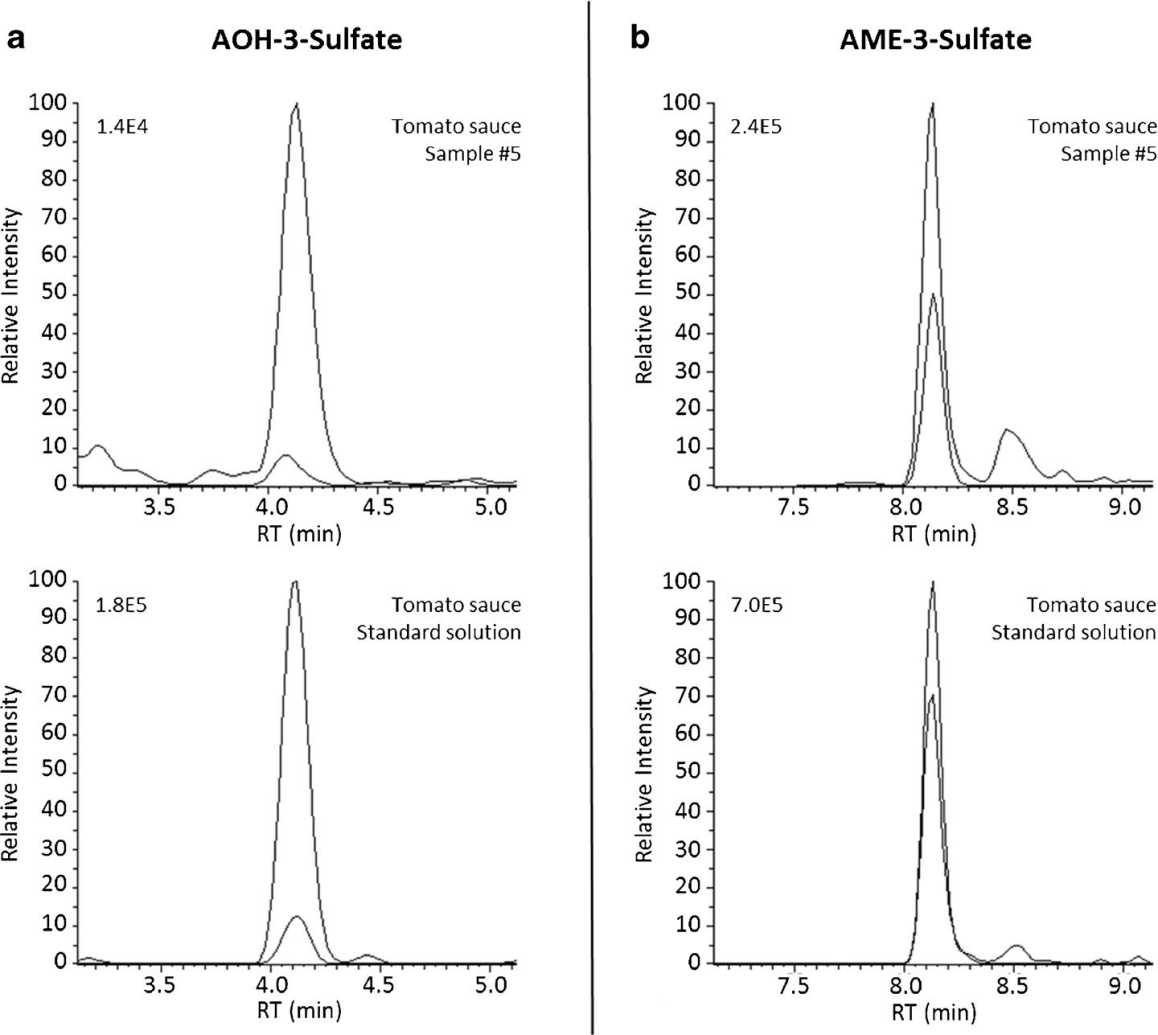
MRM-chromatograms of the modified *Alternaria* toxins AOH-3-S (**a**) and AME-3-S (**b**) in a naturally contaminated tomato sauce sample (sample #5, AOH-3-S < LOQ, AME-3-S 3.2 ng/g) compared to the respective signals in the tomato sauce matrix-matched standard solution (3 ng/mL). The chromatograms show overlaid quantifier and qualifier transitions for AOH-3-S (m/z 337 → 257 and m/z 337 → 213) and AME-3-S (351 → 256 and 351 → 271), respectively. Signals of higher intensity represent the quantifier transitions

As expected, TeA concentrations were higher than the other *Alternaria* toxins and reached concentrations of 300 ng/g. Compounds with genotoxic properties, AOH and AME, were found in about half of the tomato sauce samples. The concentrations determined are in a similar range as published in other recent studies [29, 31, 53]. To the best of our knowledge, the masked mycotoxin AOH-9-Glc was identified and quantified for the first time in any food matrix. Interestingly, only the C9 isomer conjugate was detected, despite the threefold lower LOD of AOH-3-Glc. This indicates that, at least in tomatoes, AOH-9-Glc is the prevalent formed metabolite of AOH. This is in line with in vitro studies where AOH-9-Glc was reported to be the major metabolite in tobacco suspension cell culture experiments after 48 h of AOH incubation [21]. Other recently published methods also included glucosides but did not detect them in naturally contaminated samples [29, 30, 33, 53]. Furthermore, sulfate conjugates of both AOH and AME have not been described in literature as food contaminants before. We were able to detect these compounds in naturally contaminated tomato sauce samples. For confirmation purpose, selected samples contaminated at low concentrations were enriched by a factor of five and re-measured. MRM chromatograms showing quantifier and qualifier ion transitions of AOH-3-S and AME-3-S in a naturally contaminated tomato sauce sample (sample #5) are illustrated in Fig. 3. Direct comparison to a spiked blank matrix sample allowed for unambiguous identification. Surprisingly and of relevance for risk assessment, in some samples, the modified mycotoxins were present in similar concentrations as their parent toxins (Table 3, sample #5 and #9) [21, 22]. However, these first insights suggest that glycosylation is preferred for AOH, while its monomethyl ether (AME) tends to form a sulfate conjugate. It is also possible that the sulfates are not produced by the plant but by the fungus as reported by Soukup et al. [22].

All sunflower seed oil samples were contaminated by minor amounts of TEN (< LOQ-3.4 ng/g). Major *Alternaria* toxins were detected in fewer samples and lower concentrations when compared to tomato samples or to other studies [31, 33, 37, 53]. Conjugates have not been detected in any of the sunflower seed oil and wheat flour samples. The latter matrix was generally less contaminated; only AOH and TEN were detected at levels < LOQ, and surprisingly, no TeA was detected. Other studies from China reported higher *Alternaria* toxin concentrations in wheat [38, 39]. TeA, TEN, AOH, and AME were found in 100, 97, 7, and 97% of 181 wheat flour samples, in the range of 1.76–520 ng/g, 2.72–129 ng/g, 16–98.7 ng/g and 0.32–61.8 ng/g, respectively [39].

Maximum contamination levels of *Alternaria* toxins in food and feed are currently not defined, monitored, or regulated in the European Union. According to the recent EFSA report [19, 20], this is caused by a substantial lack of occurrence and toxicity data. The method presented here clearly fulfills the requirements for contributing important information on *Alternaria* toxin contamination patterns and levels. Since the method was successfully validated and is also comparatively time- and cost-effective, it proved to be fit for the intended purpose.

## Conclusion and outlook

We report an LC-MS/MS method for the simultaneous determination of 12 parent and 5 modified *Alternaria* toxins in three food matrices frequently contaminated by these ubiquitous natural toxins, namely tomato sauce, sunflower seed oil, and wheat flour. The method was validated successfully according to the Eurachem Laboratory Guide for the validation of analytical methods [44], apart from single analytes in wheat flour showing reproducible performance, but recoveries lower than the required 70%. Overall, the method proved to be fit for purpose, its application to naturally contaminated samples. A pilot study provided first insights on *Alternaria* toxin contamination patterns and levels in food commodities purchased on the Austrian retail market. While sunflower seed oil and particularly wheat flour samples showed minor contaminations, we found five parent toxins and four modified forms of AOH and AME, partly for the first time in any food commodity, in tomato sauce samples. These results confirm that the hidden toxicological potential of masked/modified mycotoxins could be an issue and should be considered in future risk assessments. To obtain more comprehensive figures regarding occurrence patterns, follow-up large-scale surveys are required. Furthermore, the role of modified toxins and combinatory toxicological effects calls for thorough evaluation to enable proper risk assessment.

## Electronic supplementary material

ESM 1 (PDF 877 kb)

## General

Arb: Arbitrary units
ESI: Electrospray ionization
IR: Ion ratio
LC: Liquid chromatography
MRM: Multiple reaction monitoring
MS: Mass spectrometry
RT: Retention time

## ***Alternaria*** toxins

AA-III: Altenuic acid
ALT: Altenuene
ALP: Alterperylenol
AME: Alternariol monomethyl ether
AME-3-Glc: Alternariol monomethyl ether-3-glucoside
AME-3-S: Alternariol monomethyl ether-3-sulfate
AOH: Alternariol
AOH-3-Glc: Alternariol-3-glucoside
AOH-3-S: Alternariol-3-sulfate
AOH-9-Glc: Alternariol-9-glucoside
ALS: Altenusin
ATX-I: Altertoxin I
ATX-II: Altertoxin II
isoALT: Isoaltenuene
STTX-III: Stemphyltoxin III
TeA: Tenuazonic acid
TEN: Tentoxin

## Solvents and chemicals

ACN: Acetonitrile
HAc: Acetic acid
NH_4_Ac: Ammonium acetate
isoProp: Isopropanol
MeOH: Methanol
