# Supplementary material for: Tracking emerging mycotoxins in food: development of an LC-MS/MS method for free and modified Alternaria toxins
Source: Anal Bioanal Chem. 2018 May 16;410(18):4481–94. doi: 10.1007/s00216-018-1105-8 (PMC6021461; doi:10.1007/s00216-018-1105-8)
Supplement: Supplementary file 1 — (PDF 877 kb) [file 216_2018_1105_MOESM1_ESM.pdf]

Analytical and Bioanalytical Chemistry

Electronic Supplementary Material

Tracking emerging mycotoxins in food: development of an LC-MS/MS method for free and modified *Alternaria* toxins

Hannes Puntscher, Mary-Liis Kütt, Philipp Skrinjar, Hannes Mikula, Joachim Podlech, Johannes Fröhlich, Doris Marko, Benedikt Warth

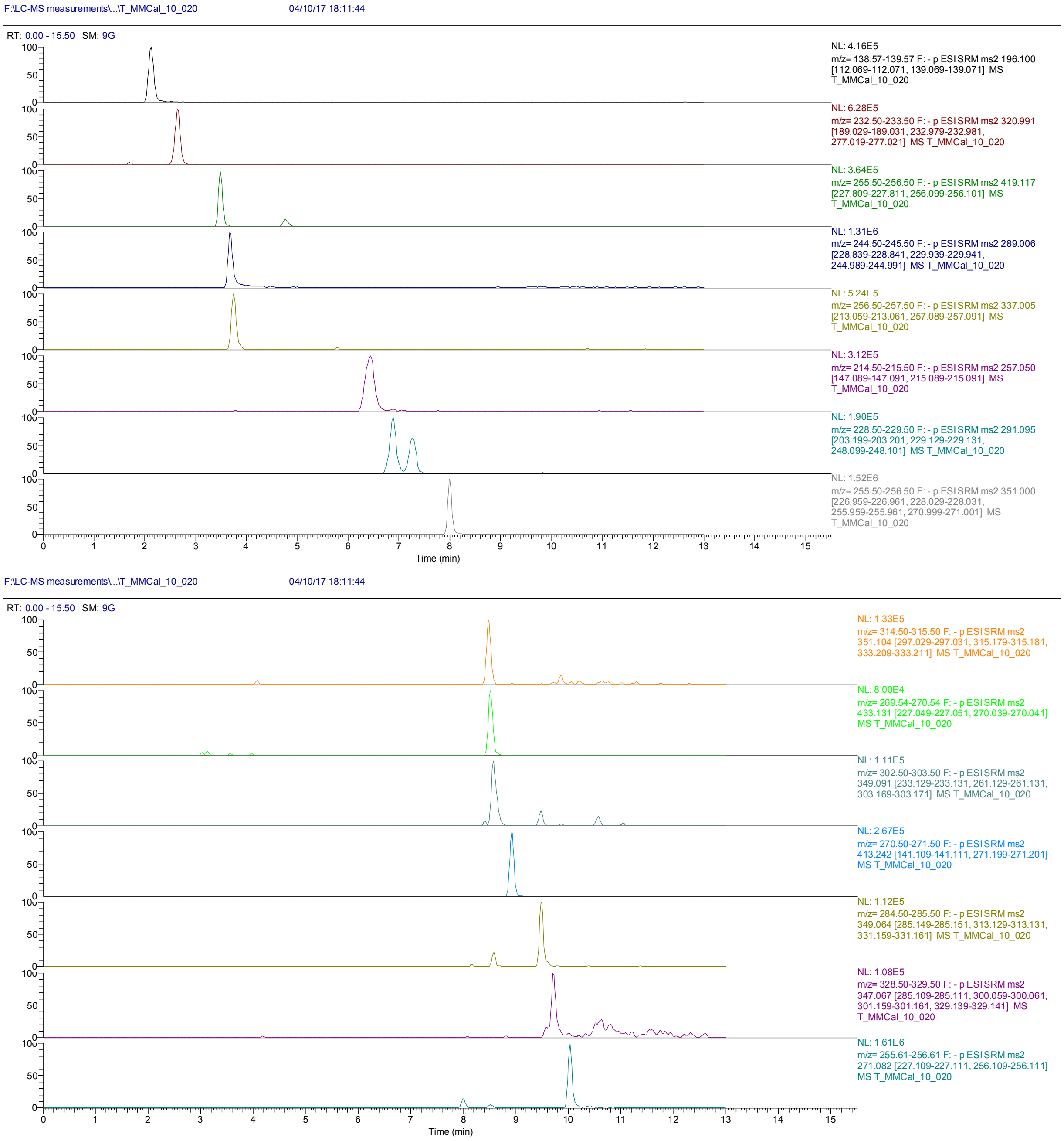

Fig. S1 Chromatographic separation of the of the 17 target analytes in tomato sauce matrix-matched calibration standards. Starting from the top: Quantifier MRMs of TeA (120 ng/mL), AA-III (20 ng/mL), AOH-3-Glc (10 ng/mL) and AOH-9-Glc (40 ng/mL), ALS (60 ng/mL), AOH-3-S (10 ng/mL), AOH (10 ng/mL), ALT (60 ng/mL) and isoALT (60 ng/mL), AME-3-S (10 ng/mL), ATX-I (2 ng/mL), AME-3-Glc (20 ng/mL), ALP (10 ng/mL), TEN (3 ng/mL), ATX-II (10 ng/mL), STTX-III (10 ng/mL), AME (2 ng/mL)

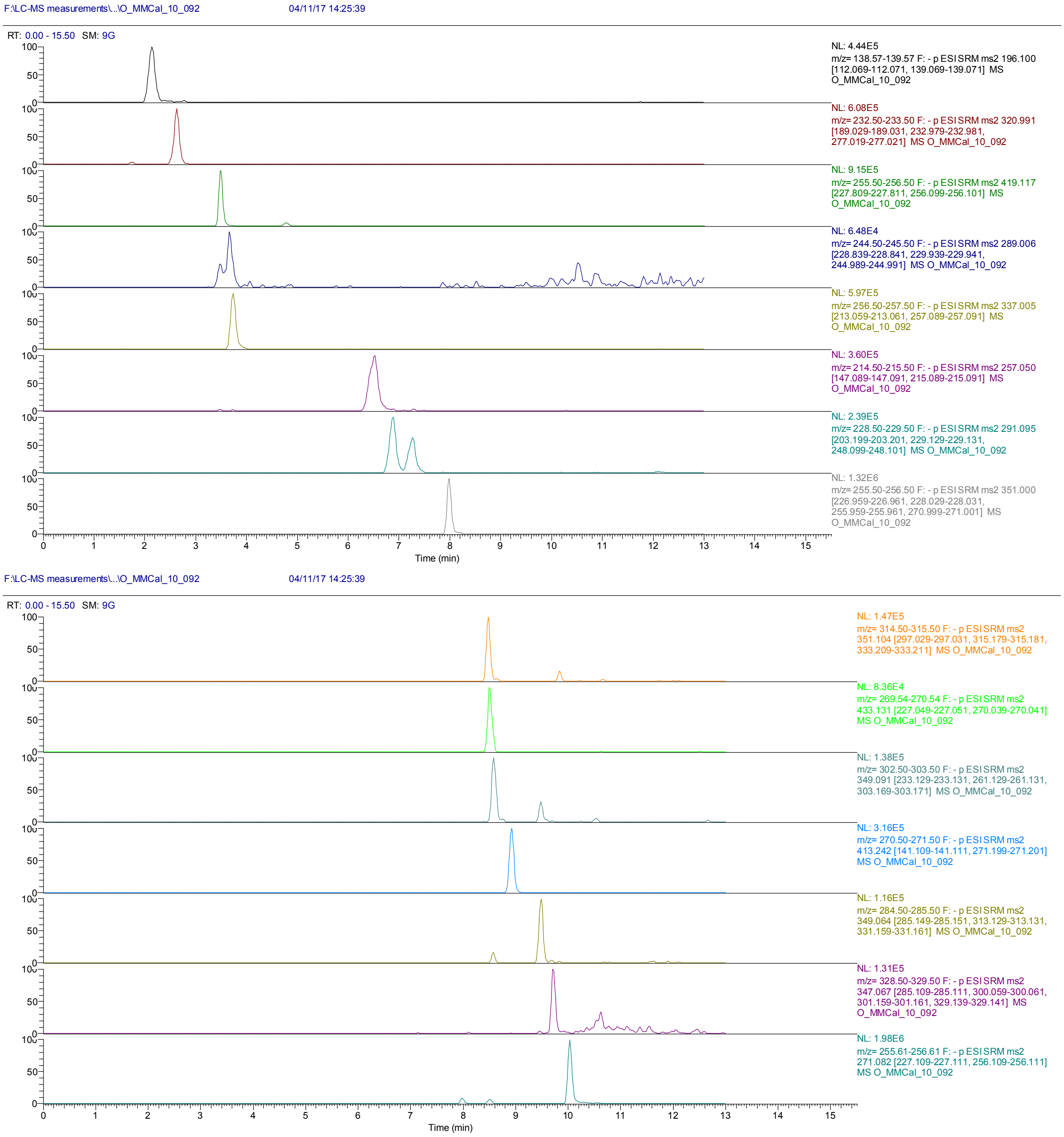

Fig. S2 Chromatographic separation of the of the 17 target analytes in sunflower seed oil matrix-matched calibration standards. Starting from the top: Quantifier MRMs of TeA (120 ng/mL), AA-III (20 ng/mL), AOH-3-Glc (10 ng/mL) and AOH-9-Glc (40 ng/mL), ALS (60 ng/mL), AOH-3-S (10 ng/mL), AOH (10 ng/mL), ALT (60 ng/mL) and isoALT (60 ng/mL), AME-3-S (10 ng/mL), ATX-I (2 ng/mL), AME-3-Glc (20 ng/mL), ALP (10 ng/mL), TEN (3 ng/mL), ATX-II (10 ng/mL), STTX-III (10 ng/mL), AME (2 ng/mL)

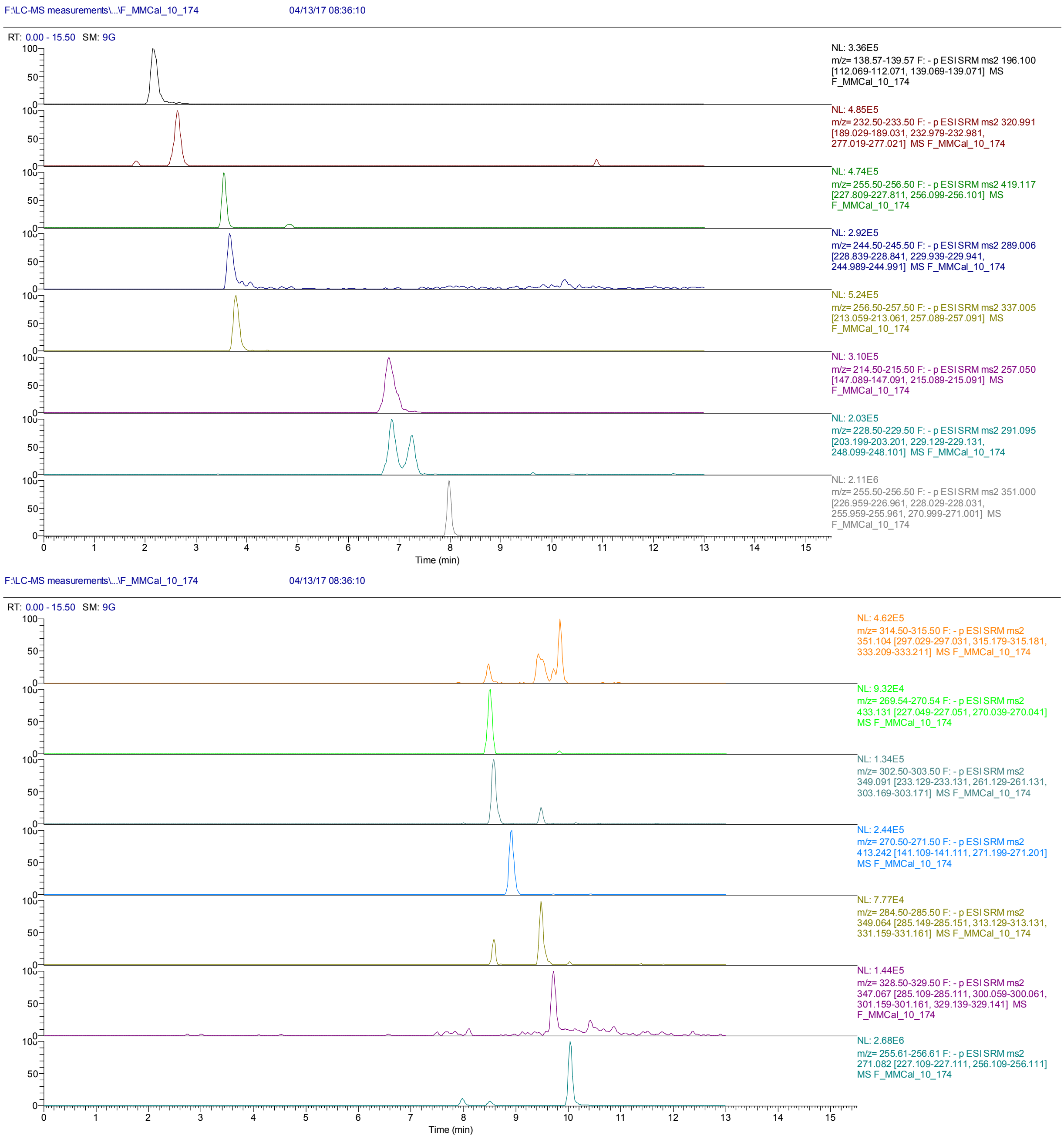

Fig. S3 Chromatographic separation of the of the 17 target analytes in wheat flour matrix-matched calibration standards. Starting from the top: Quantifier MRMs of TeA (120 ng/mL), AA-III (20 ng/mL), AOH-3-Glc (10 ng/mL) and AOH-9-Glc (40 ng/mL), ALS (60 ng/mL), AOH-3-S (10 ng/mL), AOH (10 ng/mL), ALT (60 ng/mL) and isoALT (60 ng/mL), AME-3-S (10 ng/mL), ATX-I (2 ng/mL), AME-3-Glc (20 ng/mL), ALP (10 ng/mL), TEN (3 ng/mL), ATX-II (10 ng/mL), STTX-III (10 ng/mL), AME (2 ng/mL)

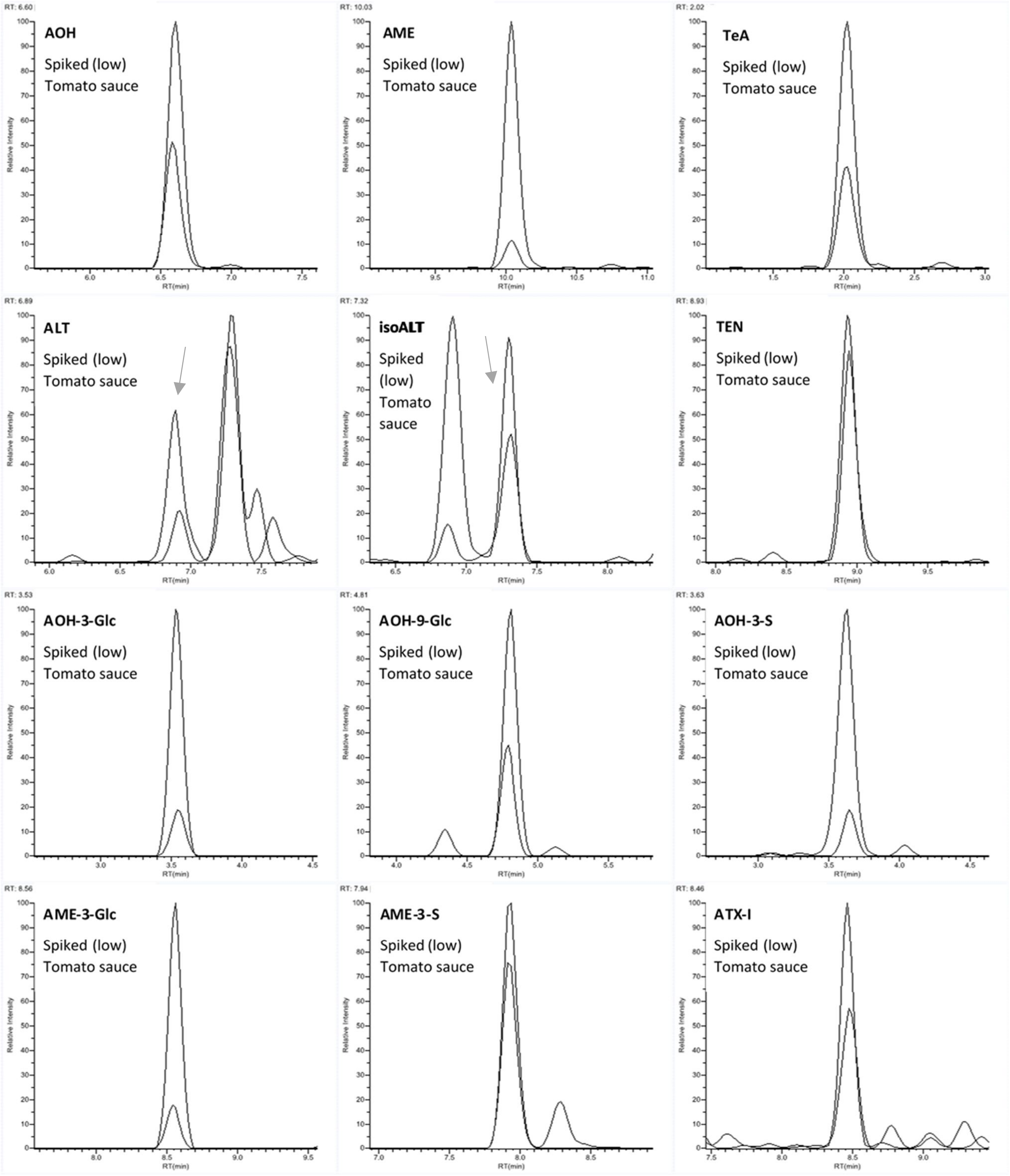

Fig. S4 continued below

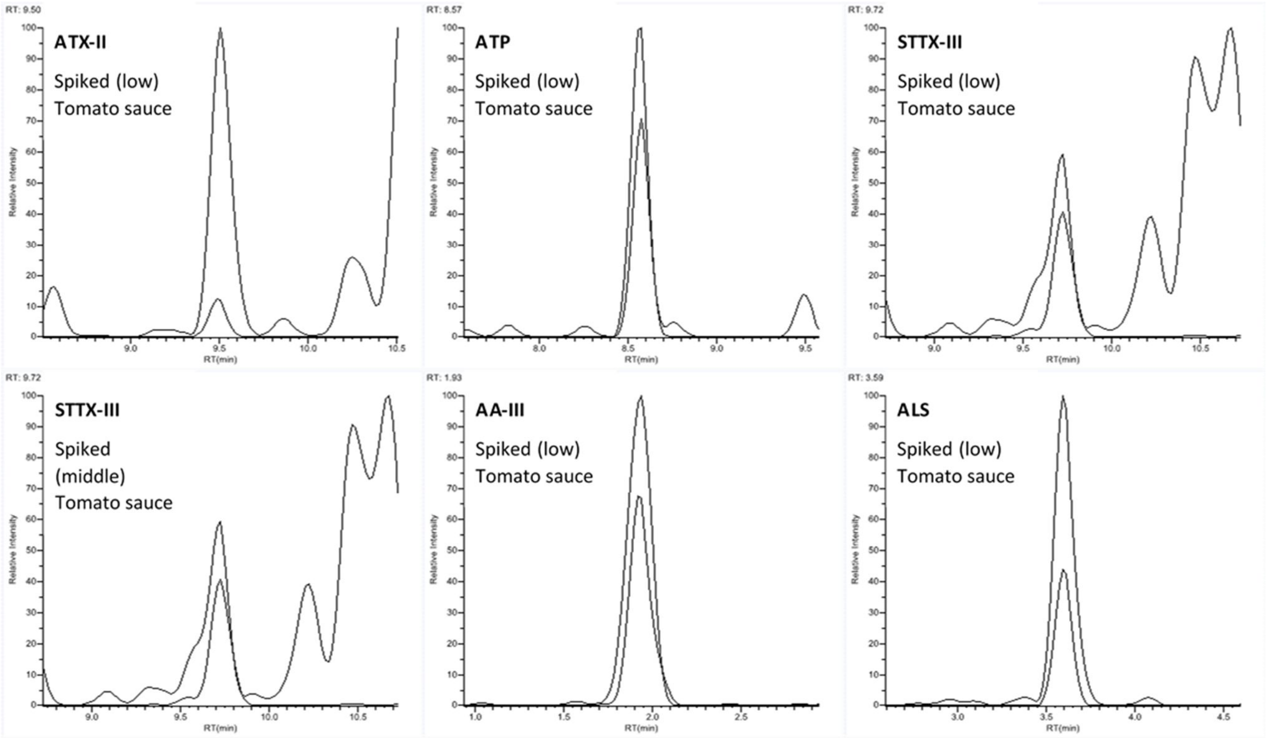

Fig. S4 Chromatographic peaks of the quantifier and qualifier ions of the 17 analytes in spiked tomato sauce (level as reported in table 2)
